# Efficacy and safety of solifenacin combined with biofeedback in children with overactive bladder

**DOI:** 10.1186/s12894-024-01486-9

**Published:** 2024-04-25

**Authors:** Yan Hu, Hui Zhang

**Affiliations:** 1 The Department of Pediatrics, West China Second University Hospital of Sichuan University, No 20 Third Section, Renmin Nan Road, Chengdu, Sichuan 610041 China; 2 Key Laboratory of Birth Defects and Related Diseases of Women and Children (Sichuan University), Ministry of Education, Chengdu, Sichuan 610041 China

**Keywords:** Overactive bladder, Solifenacin, Biofeedback, Efficacy, Side effect, Children

## Abstract

**Background:**

Overactive bladder is a common chronic urological disorder in children, liable to impact normal social activities, disrupt sleep and even impair self-esteem. We aimed to evaluate the efficacy and safety of solifenacin combined with biofeedback for paediatric overactive bladder.

**Method:**

Forty-five children with overactive bladder were enrolled and divided into three groups: 15 patients in Group A were treated with solifenacin, 15 cases in Group B with biofeedback, and the other 15 patients in Group C with the combination of solifenacin plus biofeedback. Each group was subdivided into the non-urge incontinence (non-UI) and urge incontinence (UI) groups. The remission rates were compared among the three groups at 2, 4, 8 and 12 weeks from the beginning of treatment. The side effects of solifenacin were recorded and followed up.

**Result:**

After 2 weeks since initial treatment, the complete response rates were 33.3% (5/15), 20.0% (3/15), and 53.3% (8/15) in the three groups. At 4 weeks, the complete remission rates were 46.7% (7/15), 33.3% (5/15), and 60.0% (9/15) respectively. Moreover, the complete remission rates of the UI groups were higher than the non-UI groups (*p* < 0.05). At 8 weeks, the complete response rates were 53.3% (8/15), 40.0% (6/15), and 67.7% (10/15). At 12 weeks, the complete response rates were 67.8% (10/15), 60.0% (9/15), and 86.7% (13/15). The complete response rates were higher and urodynamic parameters were improved obviously in group C than the other two groups (*p* < 0.05) during the follow-ups. The median voiding frequency decreased and median functional bladder capacity increased obviously in Group C after 4 weeks (*p* < 0.05). Dry mouth was observed in 2 patients (4.4%). 2 patients experienced constipation (4.4%), and neither case was severe. The symptoms of these four patients had relieved by reducing the dose of solifenacin.

**Conclusion:**

Solifenacin combined with biofeedback had good efficacy and compliance for children experiencing overactive bladder. It took only 2 weeks to achieve the complete response rate over 50%, especially for the improvement of UI symptoms.

## Background

Overactive bladder (OAB) is a functional disorder of the urinary bladder that is defined as ‘having urgency, usually with frequency, and with or without urge urinary incontinence’ [1, 2]. To date, OAB is considered a common disease in children that can be diagnosed on the basis of clinical symptoms. A urodynamic study is useful for elucidating the pathogenesis of OAB [3, 4].

Currently, behavioural therapy and anticholinergic drugs are at the core of treatment for OAB [5]. On the one hand, behavioural therapy is minimally invasive and does not cause adverse reactions in children. It was reported that behavioural therapy is the primary and initial treatment of the disease, including lifestyle guidance, pelvic floor exercises, biofeedback, bladder training, and toileting assistance [6]. Remarkably, biofeedback is an effective therapeutic method aimed at training contractions of the pelvic floor muscles and the degree of contraction, which can be operated via devices such as rectal manometers and electromyography [7–9]. On the other hand, drug treatment remains a very important remedy for OAB. At present, anticholinergic agents are the most commonly used with efficacy and safety [10]. Solifenacin is a new anticholinergic drug that is relatively more highly selective for the muscarinic receptor M_3_ of the bladder than for the salivary glands. This new medication has shown excellent benefits for improving symptoms of urgency, frequency, and urge urinary incontinence in adults, especially for resolution of urinary incontinence [11, 12].

Paediatric OAB would turn into a lifelong problem and it should be addressed as soon as recognized to improve the child’s symptoms, and decrease the risk of severe and refractory symptoms in their adult life [6]. Nowadays, a stepped approach was usually adopted to address this disease, beginning with behavioural therapy and progressing to anticholinergic drugs or other interventions [1]. However, behavioural therapy was considered to take effect slowly compared with anticholinergic drugs by some reports [8, 9]. Therefore, we aimed to search for an optimal treatment to get quick and effective remission for paediatric OAB. Recently, there have been some reports claiming that the combination of anticholinergic drugs plus behavioural therapy may be more effective for OAB [7, 13]. Although a consensus on the superiority of combined therapy over monotherapy has yet to be achieved, the former remains recommended for patients in some conditions [13]. Nevertheless, there are few studies about combined treatment in children with OAB. Therefore, we aimed to evaluate the efficacy and safety of solifenacin combined with biofeedback for paediatric OAB in our study.

## Method

This study was a retrospective analysis of 45 children diagnosed with OAB for the first time from June 2021 to January 2023. We randomly screened research subjects according to the pre-designed inclusion criteria, and strict exclusion criteria would increase the homogeneity of the sample. To be included, the patients had to have exhibited clinical symptoms, such as frequency or nocturia, with or without urge urinary incontinence. Moreover, the results of the urodynamic study had to have been consistent with OAB. Any children with urinary tract infection, neurogenic bladder, congenital spinal dysplasia, bladder abnormalities (such as bladder cancer, bladder calculus), or psychogenic urinary frequency were excluded. All 45 patients were divided into three groups: 15 patients in Group A were treated with solifenacin, 15 patients in Group B were treated with biofeedback, and the other 15 patients in Group C were treated with solifenacin together with biofeedback at the same time. No statistically significant differences were found among the three groups (*P* > 0.05). Each group was subdivided into non-urge incontinence (non-UI) and urge incontinence (UI) groups. All of 23 patients in three groups presented as urge urinary incontinence. The uninhibited detrusor contractions during the filling period (rise of > 15 cmH_2_O above baseline) could be found by the urodynamic studies when the patients experienced urgency. Clinical data to be collected included demographic information, lower urinary tract symptoms, and a 2-day frequency volume chart. Urinalysis, ultrasound examinations, renal function tests, uroflowmetry, urodynamic studies and residual urine checks were also performed for each child. The urodynamic detector (Laborie) was operated by the same doctor, and the results were analyzed by professional nephrologists.

All of 45 children and their parents were explained about normal lower urinary tract function and gave some life-style advices, including balanced fluid intake and diet, regular bladder emptying patterns. Meanwhile, they were instructed in behavioral modification with regular voiding habits, proper voiding posture by the specialists.

Additionally, the patients in Groups B and C received biofeedback lasting 30 min each time by means of an apparatus (Laborie), which was applied three times a week. We would provide specific guidance to every participant, especially at the beginning of biofeedback therapy. Moreover, our professional staff would accompany the patients during the entire treatment. Children were usually in a lateral position, with the myoelectric probe inserted into the anus and surface electromyography taped to the perianal skin. Then they were guided by multimedia animation images displayed on the computer screen to contract and relax the pelvic floor muscles, getting correct and effective pelvic floor muscle exercise. Computer games were fun and painless, easy to be accepted by children. Informed consent was obtained from all of the children’s parents.

Efficacy was measured on the basis of improvement in OAB subjective symptoms (assessed by OABSS), and 2-day frequency volume chart (voiding frequency, maximum voided volume) at 2, 4, 8 and 12 weeks from the beginning of treatment. Urodynamic parameters including maximum urine flow rate (Qmax), bladder sensation, bladder compliance and detrusor instability were followed up at 12 weeks. Patients were then grouped according to varying degrees of treatment response. No response was defined as < 50% reduction, partial response was defined as 50% to 99% reduction, and complete response was defined as 100% reduction [5].

The side effects of solifenacin were recorded, and these included dry mouth, constipation, and blurred vision. Then, the remission rates and incidences of side effects were compared among the three groups.

Patients in group A and group C used an adjusted-dose regimen of solifenacin (2.5–7.5 mg). Subsequent changes in dosage from initial 2.5 mg were made based on the assessment of effectiveness and safety at every 2-week follow-up, including a symptom assessment scale (Overactive Bladder Symptom Score, OABSS) and 2-day frequency volume chart. If the patients didn’t obtain the satisfactory curative effects (no response, defined as 0% to 49% reduction of symptoms), the dose of 1.25 mg was added for every two weeks until to a maximum of 7.5 mg. If the patient achieved partial response (defined as 50% to 99% reduction of symptoms) or complete response, the dose would not be adjusted. Additionally, if the side effects of solifenacin occurred, the dose would be reduced by 1.25 mg for each time, or even discontinued.

The results are expressed herein as the median, mean, and percentage. Statistical analyses were performed with SPSS software (version 18.0, SPSS), and we used the Pearson chi-square test. All reported P values < 0.05 were considered statistically significant.

## Results

**Fig. 1:**
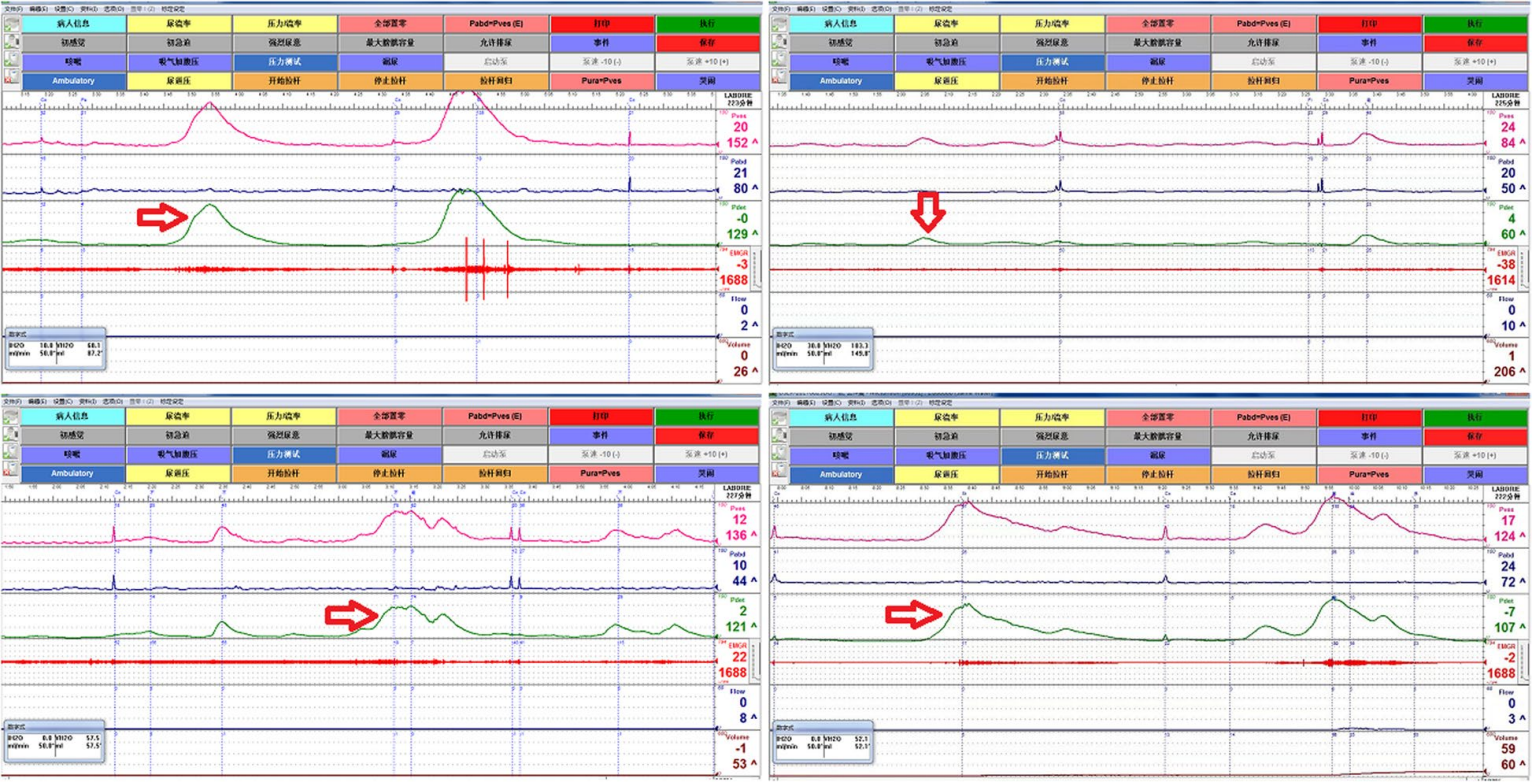
Typical uninhibited detrusor contractions in urodynamic studies of 4 patients. During the filling phase, urodynamic involuntary detrusor overactivity that may have been spontaneous or provoked and associated with urgency was found

1. Patients’ baseline characteristics
  Forty-five children (27 male, 18 female) with a median age of 7.5 years satisfied the inclusion criteria and were followed up at 2 weeks, 4 weeks, 8 weeks and 12 weeks. The median voiding frequency per day was 13 times (range 9 to 42 times), and the median functional bladder capacity was approximately 130 ml (range 70 to 180 ml). The clinical characteristics of the patients, such as age, sex, symptoms, and urodynamic study results, are shown in Table 1. The majority of patients showed typical uninhibited detrusor contractions in urodynamic studies (Fig. 1).
2. Comparison of the effectiveness among the three groups
  After 2 weeks since initial treatment, the complete response rates were 33.3% (5/15), 20.0% (3/15), and 53.3% (8/15) in the three groups, respectively. In the three groups, the median voiding frequency during the daytime decreased, and the median functional bladder capacity increased, but the differences were not statistically significant (*p* > 0.05).
  At 4 weeks, the complete remission rates were 46.7% (7/15), 33.3% (5/15), and 60.0% (9/15) in the three groups. The median voiding frequency decreased and the median functional bladder capacity increased obviously in Group C, with statistical significance compared with the other two groups (*p* < 0.05). Moreover, the complete response rates of the UI groups were higher than those of the non-UI groups (*p* < 0.05).
  At 8 weeks, the complete remission rates were 53.3% (8/15), 40.0% (6/15), and 67.7% (10/15) in the three groups. However, there was no significant difference in the remission rates between the UI and non-UI groups (*p* > 0.05). Additionally, the median functional bladder capacity of Group C still increased with statistical significance (*p* < 0.05).
  At 12 weeks, the complete response rates were 67.8% (10/15), 60.0% (9/15), and 86.7% (13/15), respectively. The most significant increase in the median functional bladder capacity was also observed in Group C (*p* < 0.05). Urodynamic parameters including maximum urine flow rate (Qmax), bladder sensation, bladder compliance and detrusor instability were improved compared with ones before treatment in Group C (*p* < 0.05). Moreover, the complete response rates were higher and urodynamic parameters were improved obviously in group C than the other two groups (*p* < 0.05) during the follow-ups (Table 2).
3. The safety of solifenacin
  During the treatment period, dry mouth as an common adverse effect was observed in 1 patient in Group A (2.2%) and 1 patient in Group C (2.2%). A total of 2 patients experienced constipation in Groups A and C (4.4%) during 12 weeks, and neither case was severe (Table 3). The symptoms of these four patients had relieved by reducing the dose of solifenacin.

**Table 1:** Clinical characteristics of 45 children with OAB

	***Group A*** ***n* = *15***	***Group B*** ***n* = *15***	***Group C*** ***n* = *15***
**Age (years)**	7.1 ± 1.4	7.9 ± 1.1	7.6 ± 0.9
**Male/female**	9 / 6	8 / 7	7 / 8
**Duration of disease (months)**	4.2	3.9	4.1
**Symptoms**			
Frequent micturition (voiding frequency > 8 times in day time and/or > 2 times at night)	15 (100.0%)	15 (100.0%)	15 (100.0%)
Urgency	15 (100.0%)	15 (100.0%)	15 (100.0%)
Urge urinary incontinence	8 (53.3%)	7 (46.7%)	8 (53.3%)
Enuresis	5 (33.3%)	6 (40.0%)	5 (33.3%)
Constipation	0	1 (6.7%)	0
**2-day frequency volume chart**			
Maximum voided volume < 65% MCC	15 (100.0%)	15 (100.0%)	15 (100.0%)
**Urodynamic study**			
Increased Qmax	12 (80.0%)	11 (73.3%)	12 (80.0%)
Increased bladder sensation	15 (100.0%)	15 (100.0%)	15 (100.0%)
Decreased bladder compliance	8 (53.3%)	9 (60.0%)	8 (53.3%)
Detrusor instability	6 (40.0%)	7 (46.7%)	7 (46.7%)

*OAB* Overactive bladder, *MCC* Maximum cystometric capacity of bladder based on age (30 + [age in years × 30] mL), *Qmax* Maximum urine flow rate

**Table 2:** Comparison of the effectiveness among three groups

	***Group A***		***Group B***		***Group C***	
	*UI group* *n* = *8*	*Non-UI group* *n* = *7*	*UI group* *n* = *7*	*Non-UI group* *n* = *8*	*UI group* *n* = *8*	*Non-UI group* *n* = *7*
**Before treatment**						
The median voiding frequency per day (times)	13	13	13	12	15	12
Median functional bladder capacity (ml)	120	130	130	140	130	120
Increased Qmax	75.0% (6/8)	85.7% (6/7)	71.4% (5/7)	75.0% (6/8)	75.0% (6/8)	85.7% (6/7)
Increased bladder sensation	100% (8/8)	100% (7/7)	100% (7/7)	100% (8/8)	100% (8/8)	100% (7/7)
Decreased bladder compliance	62.5% (5/8)	42.9% (3/7)	71.4% (5/7)	50.0% (4/8)	62.5% (5/8)	42.9% (3/7)
Detrusor instability	50.0% (4/8)	28.6% (2/7)	71.4% (5/7)	25.0% (2/8)	62.5% (5/8)	28.6% (2/7)
**At 2 weeks**						
The complete response rates	37.5% (3/8)	28.6% (2/7)	28.6% (2/7)	12.5% (1/8)	67.5% (5/8)	42.9% (3/7)
The partial response rates	37.5% (3/8)	28.6% (2/7)	28.6% (2/7)	25.0% (2/8)	25.0% (2/8)	28.6% (2/7)
The median voiding frequency per day (times)	10	10	11	10	11	9
Median functional bladder capacity (ml)	150	140	150	150	160	150
**At 4 weeks**						
The complete response rates	50.0% (4/8)	42.9% (3/7)	42.9% (3/7)	25.0% (2/8)	75.0% (6/8)	42.9% (3/7)
The partial response rates	25.0% (2/8)	28.6% (2/7)	28.6% (2/7)	37.5% (3/8)	12.5% (1/8)	42.9% (3/7)
The median voiding frequency per day (times)	10	9	10	10	8	7
Median functional bladder capacity (ml)	160	160	160	160	180	170
**At 8 weeks**						
The complete response rates	50.0% (4/8)	57.1% (4/7)	42.9% (3/7)	37.5% (3/8)	75.0% (6/8)	57.1% (4/7)
The partial response rates	37.5% (3/8)	28.6% (2/7)	42.9% (3/7)	37.5% (3/8)	25.0% (2/8)	28.6% (2/7)
The median voiding frequency per day (times)	8	8	9	9	6	6
Median functional bladder capacity (ml)	180	170	170	160	210	190
**At 12 weeks**						
The complete response rates	75.5% (6/8)	57.1% (4/7)	71.4% (5/7)	50.0% (4/8)	87.5% (7/8)	85.7% (6/7)
The partial response rates	12.5% (1/8)	42.9% (3/7)	14.3% (1/7)	25.0% (2/8)	12.5% (1/8)	14.3% (1/7)
The median voiding frequency per day (times)	6	7	7	8	5	6
Median functional bladder capacity (ml)	200	190	190	180	240	220
Increased Qmax	25.0% (2/8)	28.6% (2/7)	28.6% (2/7)	25.0% (2/8)	12.5% (1/8)	14.3% (1/7)
Increased bladder sensation	25.0% (2/8)	28.6% (2/7)	42.9% (3/7)	37.5% (3/8)	25.0% (2/8)	14.3% (1/7)
Decreased bladder compliance	12.5% (1/8)	14.3% (1/7)	28.6% (2/7)	25.0% (2/8)	0	14.3% (1/7)
Detrusor instability	0	0	14.3% (1/7)	12.5% (1/8)	0	0

*OAB* Overactive bladder, *UI* urge incontinence

**Table 3:** The side effects of solifenacin in group A and group C

	***Group A***		***Group C***	
	*UI group* *n* = *8*	*Non-UI group* *n* = *7*	*UI group* *n* = *8*	*Non-UI group* *n* = *7*
**At 2 weeks**				
Dry mouth	0	0	0	0
Constipation	0	0	0	0
Blurred vision	0	0	0	0
**At 4 weeks**				
Dry mouth	0	0	0	0
Constipation	0	0	0	0
Blurred vision	0	0	0	0
**At 8 weeks**				
Dry mouth	1 (12.5%)	0	0	0
Constipation	0	0	1 (12.5%)	0
Blurred vision	0	0	0	0
**At 12 weeks**				
Dry mouth	0	0	0	1 (14.3%)
Constipation	0	1 (14.3%)	0	0
Blurred vision	0	0	0	0

*UI* urge incontinence

## Discussion

OAB is a common chronic urological disorder, with an incidence in school-aged children ranging from 17.8% to 26% [6, 14]. In addition, this condition is liable to impact normal social activities, disrupt sleep and even impair self-esteem, which extremely affects patients’ quality of life. Most urologists take a stepped approach to address this disease, beginning with the least invasive therapy (lifestyle guidance, pelvic floor exercises, biofeedback, bladder training) and progressing to more invasive or costly interventions (anticholinergic drugs, neuromodulation, surgery) [1, 15].

Biofeedback is a form of re-education or learning in which the patient is retrained within a closed feedback loop [7]. Information related to the participant's normally unconscious physiologic processes is presented as a visual, auditory or tactile signal. Biofeedback improves the contractile function of urethral sphincter and anal levator. At the same time, the neuromuscle and afferent nerve are stimulated, coupled with the repeated movement pattern information introduced into the central nervous system, gradually restoring the motor function. Indeed, in recent studies, biofeedback has been successfully applied in cases of urinary incontinence due to detrusor instability, with a reduction in morbidity and adverse effects [13, 16]. However, patients need to be intelligent enough to understand what is expected of them during the operating process. Additionally, biofeedback can be an adjunct to other forms of treatment, such as anticholinergic drugs, and is particularly useful in children [16].

For adult patients, various drugs, such as oxybutynin, tolterodine and solifenacin, have been introduced and used widely with proven efficacy and safety [17–19]. In contrast, the drugs available to children are limited. Data about the efficacy and safety of newer anticholinergic drugs in children are scarce [10]. As a consequence, the management of paediatric OAB is still considered to be challenging and complex. To date, only oxybutynin has been officially approved for children by medical authorities in North America [10, 19, 20]. Solifenacin has been accredited by the Food and Drug Administration (FDA) for OAB in adults since 2005. It has a long half-life, excellent bioavailability, and is highly selective for the muscarinic receptor M_3_ of the bladder than for the salivary glands [11, 12]. Some studies have indicated that the affinity of Solifenacin to M_3_ receptor is about 14.2 times higher than that of M_2_ receptor, while the affinity of tolterodine to M_3_ receptor is almost no difference from M_2_ receptor. Therefore, the incidence of dry mouth, the greatest problem with anticholinergic drugs, was lower in solifenacin group than tolterodine [21–23]. Oxybutynin is one of the most widely used M-receptor antagonists for children with OAB, but many children have experienced unbearable complications (constipation, dry mouth, blurred vision, headaches, flushing of the face, abnormal behavior). There are some reports regarding the side effects of central nervous system, such as cognitive impairment [11, 19, 24]. Two open-label, baseline-controlled, phase 3 studies were conducted in pediatric patients aged 6 months to 18 years with neurogenic detrusor overactivity, who were treated with sequential doses of solifenacin over 40-week treatment period. This study concluded that solifenacin was effective and well tolerated, suggesting this medicine may be a viable alternative to oxybutynin for children [25]. Hoebeke and colleagues performed a retrospective uncontrolled study of 138 children with OAB who were treated with solifenacin for a mean of 23 months. They found solifenacin to be effective with an overall 85% response rate and side effects in only 6.5% of their cohort [12]. A long-term study conducted in Japan enrolled 252 OAB patients, in which treatment was continued for 52 weeks (or 60 weeks), suggesting that solifenacin is a safe drug that could be taken continuously [26]. In our study, side effects were observed in only 4 patients (8.9%), and none of them experienced severe symptoms.

To our knowledge, some studies have shown that the combination of anticholinergic drugs and biofeedback is the most effective approach for adults [7, 13]. Nevertheless, few studies on combined treatment in children have been reported. With this in mind, we conducted a study of solifenacin plus biofeedback applied for paediatric OAB. The subjective perceived benefit and overall satisfaction were better for patients in Group C (combination therapy). We found that the combination therapy led to a significant decrease in voiding frequency and an increase in functional bladder capacity. Moreover, this treatment dramatically improved lower urinary tract symptoms (LUTS), especially in the UI group, with a complete response rate of 87.5% at 12 weeks. According to our results, the combination treatment took only 2 weeks to achieve a complete response rate exceeding 50%. Currently, there was no unified standard for the treatment course of solifenacin or feedback. As a whole, clinical judgement remains paramount to individualize such an approach.

We believe that our study will provide advantageous evidence on the efficacy and safety of the combination of solifenacin with biofeedback for paediatric OAB in clinical practice. However, further randomized controlled studies would be required to obtain official approval and recommend this combination therapy for routine use in children.

## Conclusion

Solifenacin combined with biofeedback had good efficacy and compliance as an available cure for children experiencing OAB with or without UI. It took only 2 weeks of treatment to achieve the complete response rate over 50%, especially for the improvement of UI symptoms. We believe that such combination therapy deserves to be extensively adopted clinically.

## Data Availability

All data generated or analyzed during this study are included in this article.

## Abbreviations

OAB: Overactive bladder
UI: Urge incontinence
FDA: Food and Drug Administration
LUTS: Lower urinary tract symptoms
OABSS: Overactive Bladder Symptom Score
Qmax: Maximum urine flow rate
